# Supplementary material for: The causal relationship between the human gut microbiota and pyogenic arthritis: a Mendelian randomization study
Source: Front Cell Infect Microbiol. 2024 Nov 26;14:1452480. doi: 10.3389/fcimb.2024.1452480 (PMC11629706; doi:10.3389/fcimb.2024.1452480)
Supplement: Supplementary file 1 [file DataSheet1.zip › mendelian test/ebi-a-GCST90027651.csv_forest.pdf]

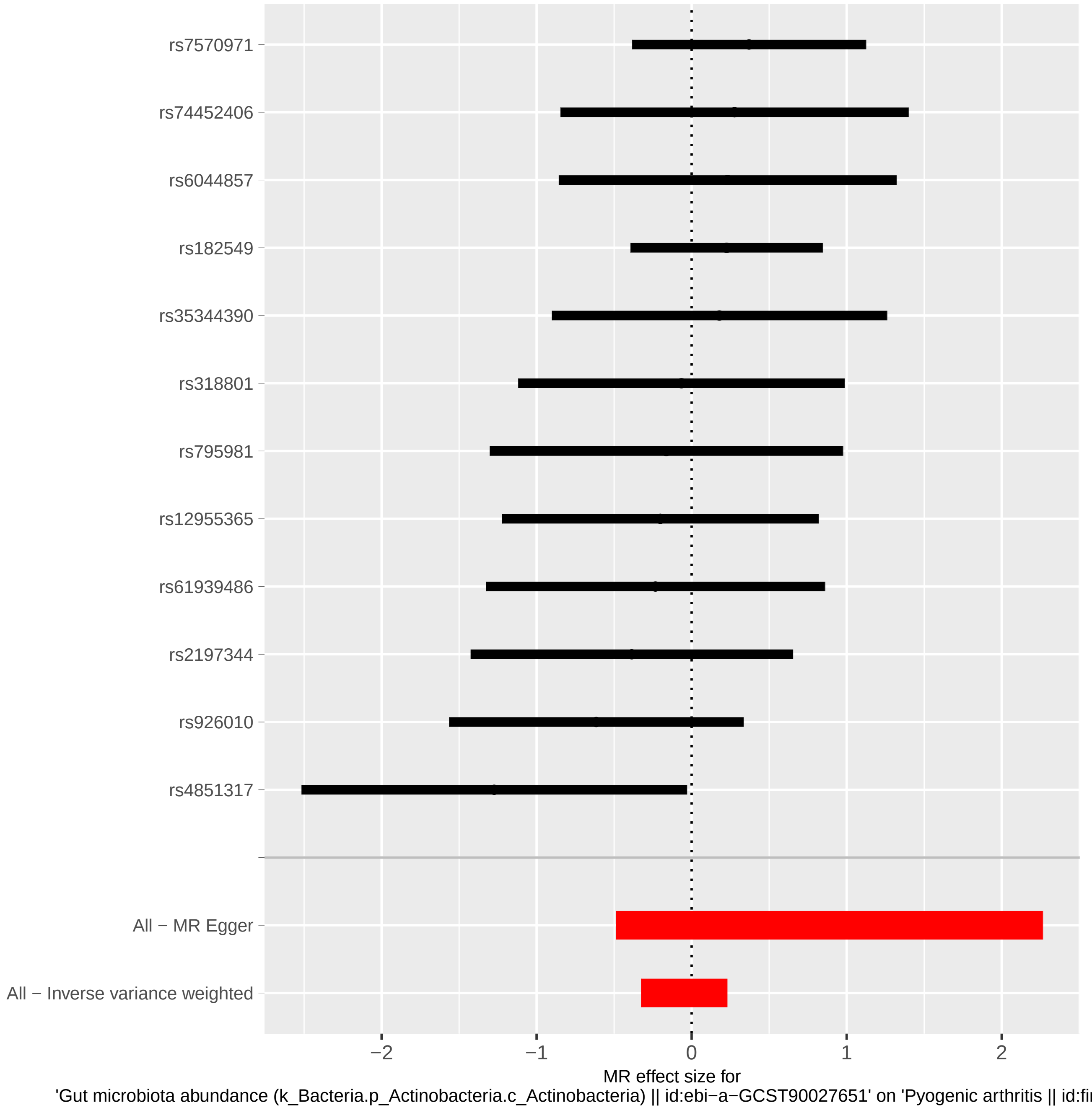
rs7570971
rs74452406
rs6044857
rs182549
rs35344390
rs318801
rs795981
rs12955365
rs61939486
rs2197344
rs926010
rs4851317
All − MR Egger
All − Inverse variance weighted
−2
−1
0
1
2
MR effect size for
'Gut microbiota abundance (k_Bacteria.p_Actinobacteria.c_Actinobacteria) || id:ebi−a−GCST90027651' on 'Pyogenic arthritis || id:fi
